# Emergence Profile Angle Matters—Restoring Peri‐Implant Health by Adjusting Prosthetics—A Narrative Review

**DOI:** 10.1111/jerd.70109

**Published:** 2026-01-30

**Authors:** Miha Pirc, Jonathan Esquivel, Andrea Patrizzi, Ronald E. Jung, Franz J. Strauss

**Affiliations:** ^1^ Clinic of Reconstructive Dentistry, Center for Dental Medicine, University of Zurich Zurich Switzerland; ^2^ Private Practice Prosthodontist Metairie Louisiana USA; ^3^ Universidad Autonoma de Chile Santiago Chile

**Keywords:** dental implant, emergence profile, prosthodontics

## Abstract

**Objective:**

To review the biological and clinical relevance of prosthetic design, specifically the emergence profile and restorative angle in influencing peri‐implant tissue health, and to illustrate how biologically driven prosthetic modifications can resolve inflammation and support peri‐implant stability.

**Overview:**

This narrative review synthesizes current evidence on the anatomy and function of the implant supracrestal complex, focusing on how restorative contour, abutment height, and emergence geometry impact soft tissue adaptation and marginal bone maintenance. It integrates recent findings from histological, clinical, and preclinical studies and highlights the role of CAD/CAM workflows in achieving biologically favorable restorations. A clinical case report illustrates the correction of peri‐implant inflammation and bone loss through the redesign of a concave, polished emergence profile on a taller titanium base.

**Conclusions:**

Restorative angle and emergence profile are critical, modifiable elements of implant‐supported prostheses. When designed in accordance with biological parameters, they contribute significantly to tissue health and long‐term esthetic outcomes. Suboptimal designs may promote plaque accumulation and inflammation, even in compliant patients. Biologically oriented, digitally guided prosthetic designs represent a key strategy in preventing peri‐implant disease.

**Clinical Significance:**

Long‐term peri‐implant health depends not only on surgical accuracy but also on biologically driven prosthetic design. Contours that respect the dimensions of the soft tissue complex, particularly concave, polished emergence profiles supported by adequately tall abutments, promote soft tissue stability, facilitate hygiene, and minimize the risk of inflammation or recession. While digital workflows offer precision in prosthetic design, clinicians must still apply sound biological principles and clinical judgment when planning and executing implant‐supported restorations. A biologically driven approach, based on a clear understanding of peri‐implant anatomy and guided by common sense, is essential for achieving predictable and esthetically successful outcomes.

## 1 Introduction

Planning, placing, and restoring dental implants requires a proper synergy between the restorative dentist, the surgeon and the dental laboratory team [1]. Each member of the implant team plays a distinct and critical role in influencing the long‐term biological and esthetic outcomes of implant‐supported rehabilitations. Key parameters such as implant position, peri‐implant soft tissue quality, and abutment design must be carefully considered and harmonized to achieve predictable success.

When designing an implant restoration, the transmucosal region connecting the implant and prosthesis, often termed the emergence profile (EP), is increasingly recognized as a key determinant of peri‐implant tissue health and stable clinical outcomes [2]. The EP describes the prosthetic contour of the transmucosal portion of the restoration (i.e., the restoration/abutment geometry as it transitions from the implant platform to the cervical crown form). Conversely, the transmucosal tunnel is an anatomic soft‐tissue corridor that forms around (and is shaped by) the implant–abutment–restoration complex and is exposed to a microbially dynamic environment.

**FIGURE 1:**
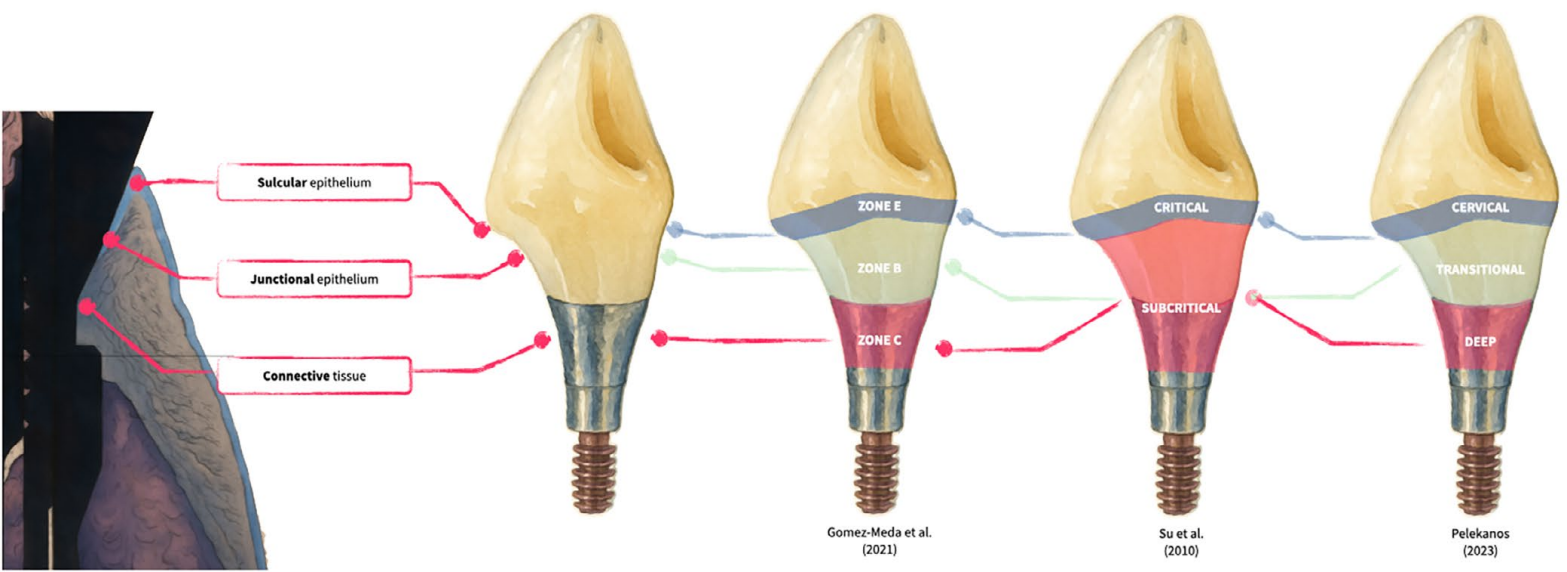
Schematic representation of various nomenclatures used to describe the implant supracrestal complex. The illustration integrates the Esthetic Biological Contour (EBC) concept proposed by Gómez‐Meda et al. [6] and Esquivel et al. [7], the “critical and subcritical contour” model described by Su et al., and the tripartite classification of cervical, transitional, and deep zones introduced by Pelekanos et al. These overlapping frameworks are presented in relation to the underlying biological structure, including the sulcular epithelium, junctional epithelium, and connective tissue, to provide a unified interpretation of how restorative design corresponds to peri‐implant soft tissue architecture.

To describe the peri‐implant soft‐tissue compartment located coronally to the implant platform, contemporary literature has introduced related concepts, including supracrestal tissue height (STH) [3], “Implant Supracrestal Complex” (ISC) [4] and “Supraplatform Complex” [5]. Although terminology varies, these concepts emphasize the functional and biological relevance of the peri‐implant soft‐tissue seal (Figure 1).

Prosthetic design serves both esthetic and biological functions. Esthetically, the EP supports a natural transition from the implant platform to the cervical crown form. Biologically, transmucosal contour, restorative angle, and the position of restorative margins influence biofilm retention, maturation of peri‐implant mucosa, establishment of a biological seal, and marginal bone stability [8, 9]. Observational studies and cross‐sectional analyses have associated unfavorable prosthetic contours and excessive restorative angles with peri‐implant inflammation [10, 11] and peri‐implantitis [12, 13]. More recently, randomized trials have evaluated the impact of abutment and emergence morphology on peri‐implant mucosal margin stability [14, 15, 16, 17].

The stability of the peri‐implant soft‐tissue complex is inherently multifactorial. In addition to mucosal thickness, implant surface characteristics and macro‐design, surgical approach, and three‐dimensional implant positioning, other relevant modifiers include the width and quality of keratinized mucosa, abutment material, shape and height, and the design of the implant–abutment and restorative interfaces (e.g., platform switching, connection type, and the position of the restorative microgap).

The purpose of this article is to illustrate how biologically driven prosthetic design can improve peri‐implant tissue stability, reduce biological complications and serve as a clinical aid for the implant restorative team.

## 2 Biological Basis of the Peri‐Implant Mucosa

Similar to natural teeth, the peri‐implant mucosa comprises sulcular and junctional epithelium with an underlying connective tissue zone. Histologic data indicate an epithelial adhesion of around 2 mm [18] and a connective tissue band of 1.5–2 mm, forming a vertical dimension of roughly 3.5–4 mm above the implant platform [18, 19]. This “supracrestal complex” acts as a protective barrier for the underlying bone. Although the overall organization resembles that of natural teeth, key structural differences exist. Histological studies have shown that collagen fibers in peri‐implant connective tissue run parallel to the implant surface and lack direct attachment, unlike the perpendicular anchorage into cementum at teeth [20]. The absence of the periodontal ligament also reduces vascularization; thus, blood supply derives mainly from supraperiosteal vessels [21]. The epithelial barrier at implant sites also differs structurally, exhibiting increased permeability and a connective tissue zone characterized by a higher proportion of collagen and fewer fibroblasts [22]. These anatomical and vascular distinctions make peri‐implant tissues more susceptible to inflammation and bone resorption when challenged by biofilm [23]. Maintaining adequate soft tissue volume and integrity is therefore critical for preserving the biological seal and long‐term stability.

## 3 Implant Position and Its Repercussion on Emergence Profile Geometry

Proper implant positioning is a cornerstone of biologically and esthetically successful implant‐supported restorations. Four key spatial parameters must be considered during treatment planning: implant depth, interproximal (mesio‐distal) position, bodily (bucco‐oral) position, and axial inclination. Each dimension has a direct impact on the geometry of the EP and, consequently, on the biological behavior and esthetic outcome of peri‐implant tissues [1, 24].

Most studies support a concave EP as the preferred prosthetic design [14, 15, 25, 26, 27, 28]. However, a flat or convex EP may be indicated in selected cases, such as when implants are placed too palatally or lingually, or show unfavorable angulation [29, 30], particularly in the coronal part of the transmucosal zone near the marginal mucosa [31]. Limited convexity may also help mask small soft‐tissue deficiencies (e.g., ridge concavities) or intentionally move the mucosal margin apically [26]. This approach should be used cautiously, as excessive convexity may increase the risk of further soft‐tissue recession. Clinical experience suggests that combining a concave B‐zone (subcritical contour) with a slightly convex E‐zone (critical contour) may help better control peri‐implant soft‐tissue recession [2, 7, 24, 32].

### 3.1 Implant Depth

Depth is among the most crucial dimensions which will influence prosthetic design. It has been described that implants should be placed 3–5 mm apical of the future restorative zenith point to allow for a proper abutment design [24]. Shallow placement may force the prosthetic flare to approximate the crestal bone, potentially resulting in marginal bone remodeling. However, deeper placement elongates the mucosal tunnel, potentially compromising oxygen diffusion and biofilm control and delaying the resolution of inflammation [11]. Tunnels exceeding 3 mm have been associated with elevated plaque accumulation, delayed inflammation resolution, and higher bleeding scores—even in patients under regular maintenance care [11, 33]. Therefore, implant depth must balance emergence geometry with hygiene accessibility.

The choice of prosthetic components is also influenced by implant depth. Stock abutments, though convenient, are often limited in gingival heights and may be unsuitable for subcrestal implants, as they may place the restorative margin within the connective tissue zone [19]. Taller titanium bases (≥ 2 mm) relocate the restorative interface coronally, preserving the biological seal and promoting crestal bone stability [7, 34, 35].

### 3.2 Interproximal (Mesio‐Distal) Position

The interproximal position of the implant can influence abutment morphology and tissue response. When implants are placed too mesially or distally, the EP tends to flatten on the offset side, compromising space for both prosthetic materials and soft tissues. This limitation may restrict the clinician's ability to use prefabricated components such as titanium bases. Additionally, a lack of concavity in interproximal regions can reduce the volume of soft and hard tissue, potentially leading to compromised papillae or bone peak resorption.

### 3.3 Bodily (Bucco‐Oral) Position

Biologically favorable concave profiles require adequate spatial conditions, particularly in the bucco‐lingual dimension. If the implant is positioned too far buccally, it restricts the space available for both restorative material and soft tissue, often resulting in a flat EP. This not only compromises esthetics but may necessitate a switch from zirconia to titanium abutments due to minimal material thickness requirements. Titanium abutments may be used without esthetic compromise if tissue thickness exceeds 3 mm; however, a buccally positioned implant inherently reduces this thickness and limits the tissue's ability to mask the abutment, thereby increasing the risk of esthetic compromise [36, 37]. Furthermore, buccal malpositioning has been linked to a threefold increase in the risk of facial bone dehiscence compared to implants placed within ideal prosthetic envelopes [38].

### 3.4 Axial Inclination

Axial inclination of the implant plays a significant role in determining the geometry of the EP. While modern restorative protocols—such as the use of dynamic screws and angulated screw channels—can compensate for implant angulations of up to 28°, these solutions primarily address prosthetic access, not the biological impact of implant positioning. Excessive facial inclination often results in the implant neck violating the space for the facial bone, potentially compromising bone volume and soft tissue thickness. This may lead to biologic complications, including recession or crestal bone remodeling. Additionally, steep axial inclinations inherently produce flatter EPs, limiting the restorative benefit associated with concave transmucosal contours. As a result, the potential to support soft tissue maturation and reduce inflammation through prosthetic design is diminished.

If a large angle correction is needed then, the screw chimney will increase in size and be shifted apically, thus approximating the flare of the restoration to the crest of the bone. Fabrication of custom abutments that allow the use of dynamic screws is a possibility to reduce the dimensions of the implant abutment in the C‐zone. However, to maintain a slim C‐zone these abutments will only have the capacity to correct small off‐axial placement of the implant (access hole in the incisal edge or slightly facial to it).

## 4 Relation of Abutment Geometry and Peri‐Implant Stability

The geometry of the EP, its contour, and restorative angle, play a pivotal role in soft‐tissue adaptation. Narrow restorative angles, typically associated with concave profiles, favor coronal tissue migration and a well‐organized epithelial cuff [9, 20, 39]. In contrast, wider or convex contours reduce the space for tissue maturation, increasing the risk of mucosal recession and inflammation [14, 26]. The literature has reported increased bone remodeling ad excessive flare of abutments approximate to the bone crest [40]. Thus, transition from implant platform to prosthesis should be carefully designed. The apical 1–2 mm above the platform, which is mainly connective tissue [18], should remain concave or straight to accommodate the STH [41] and promote crestal bone stability [40, 42]. Coronally, the contour can widen gradually to mimic natural tooth anatomy while maintaining an emergence angle below < 30° [12] < 40° [9] for optimal tissue adaptation and plaque control.

### 4.1 Design Considerations

Prosthetic design is dictated by implant position and available soft tissue. Implant position is among the most important considerations, as it will directly influence prosthetic design, including material selection, space for hard and soft tissues, and the biological response of these tissues to volume reduction [9, 31, 40]. To minimize these risks, careful preoperative planning is essential and should include guided surgery, adjunctive procedures such as hard‐ and soft‐tissue grafting, and appropriate provisionalization at the time of implant placement.

### 4.2 Concave Versus Convex

Prosthetic design within the implant supracrestal complex strongly influences plaque accumulation and inflammation. While the microbial etiology of peri‐implant diseases is well known, recent studies underscore the role of prosthetic geometry in modulating microbial retention. Wider emergence angles have been correlated with greater plaque accumulation and inflammation, independent of patient factors [11].

Convex profiles restrict soft‐tissue maturation and hinder effective cleaning, whereas concave contours allow tissue proliferation and vascularization, improving resistance to bacterial insult. These design parameters affect not only esthetics but also the capacity to maintain an intact epithelial barrier [9]. A recent randomized controlled trial demonstrated that implants restored with concave profiles had significantly lower rates of mid‐facial mucosal recession (13.3%) compared to convex profiles (46.7%) at 3 years, underscoring the clinical relevance of prosthetic contouring [15]. Over‐contoured or rough transmucosal surfaces act as plaque‐retentive niches that accelerate mucosal inflammation and barrier breakdown.

Recent data further support the relevance of surface characteristics in modulating peri‐implant tissue responses, however, they also highlight important limitations of increased roughness in the transmucosal zone. Although moderately roughened titanium surfaces (~0.95 μm) were associated with increased collagen production and a shift toward an anti‐inflammatory macrophage profile in vitro, fibroblast adhesion was reduced on rougher surfaces compared with machined controls, indicating a less stable soft tissue attachment. From a clinical perspective, reduced fibroblast adhesion combined with increased surface roughness may favor plaque retention, thereby outweighing potential biological benefits and reinforcing the preference for smooth, highly polished transmucosal surfaces to support long‐term peri‐implant health [43].

**FIGURE 2:**
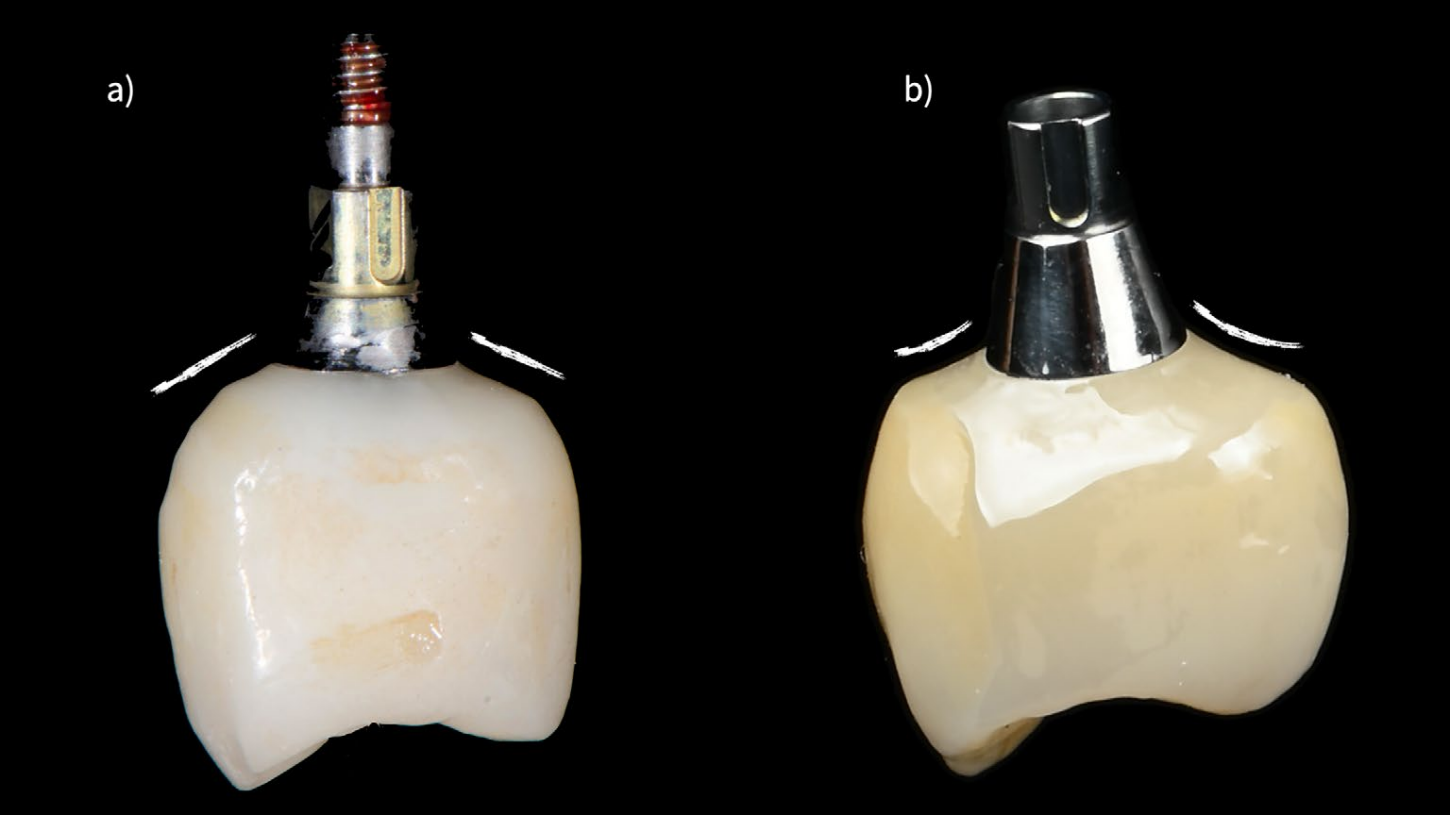
(a) Initial crown featuring a convex emergence profile and a short titanium base abutment; (b) new crown exhibiting a concave emergence profile designed on a higher titanium base abutment.

**FIGURE 3:**
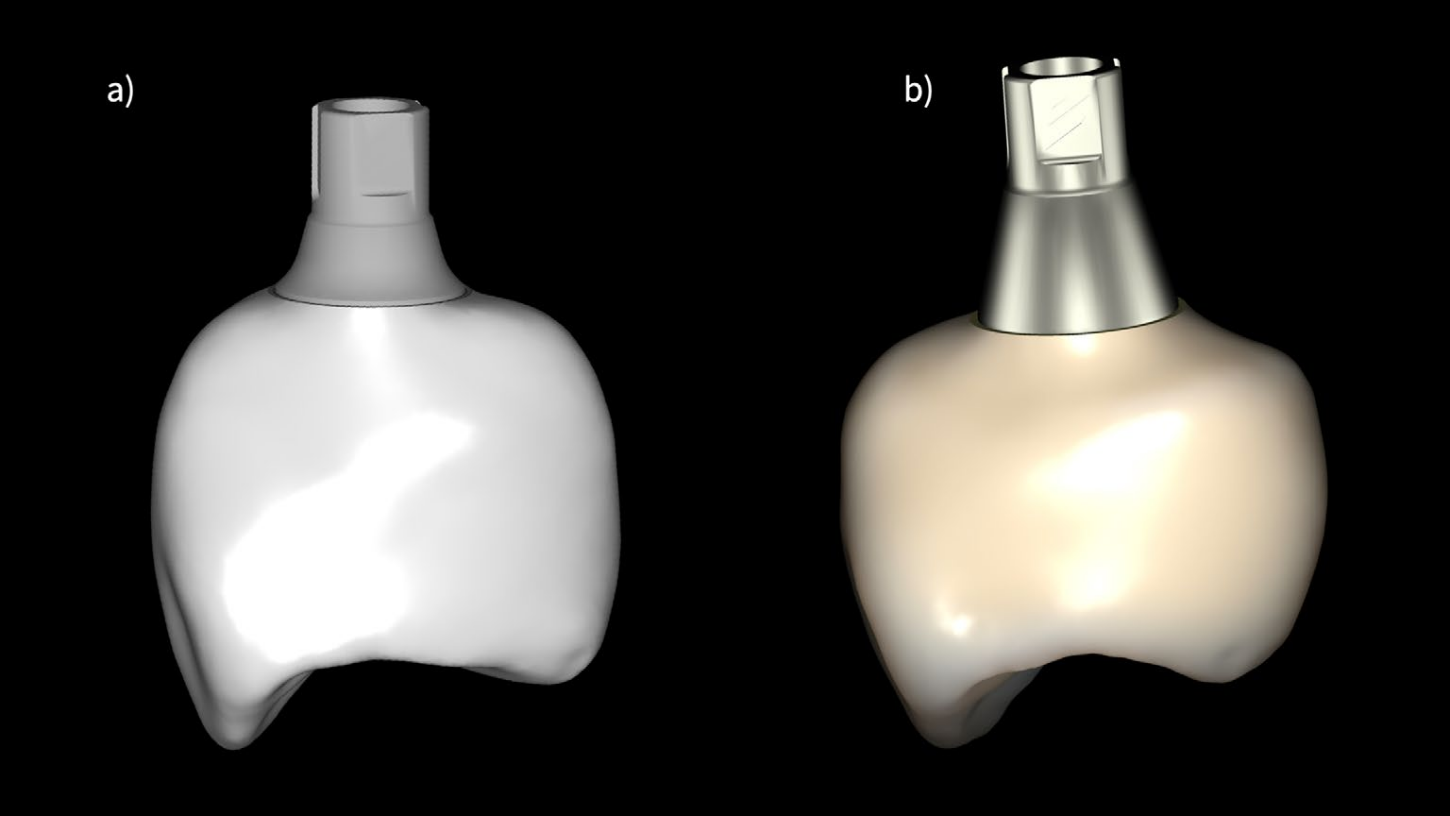
Digital representation and design comparison of: (a) the original crown with a convex emergence profile and short titanium base; (b) the new crown with a biologically optimized concave emergence profile and a higher titanium base abutment.

**FIGURE 4:**
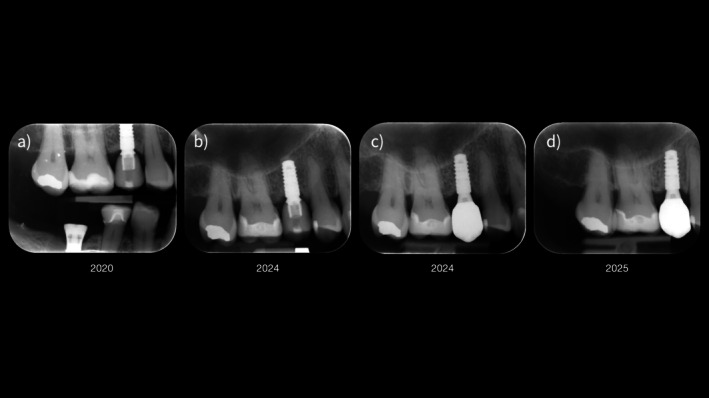
Sequential periapical radiographs illustrating changes over time: (a) Baseline image following insertion of a lithium disilicate crown on a short titanium base abutment; (b) radiograph 4 years postloading showing an angular defect and marginal bone loss at the mesial aspect of the implant; (c) timepoint of insertion of a monolithic zirconia crown on a higher titanium base abutment; (d) 4‐month follow‐up revealing stable marginal bone levels and radiographic resolution of the angular defect.

**FIGURE 5:**
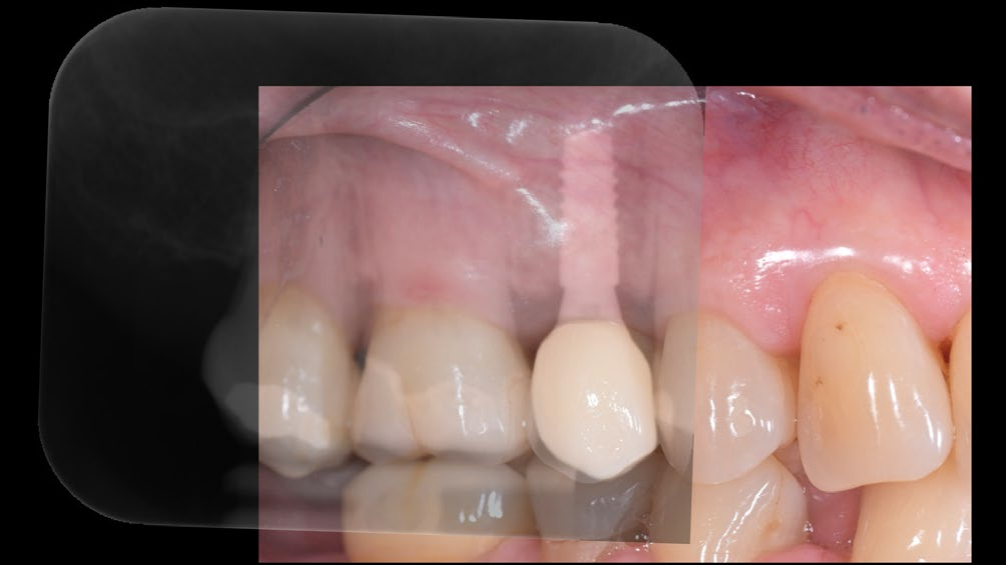
Intraoral photograph of the revised restoration at the 4‐month follow‐up, with radiographic superimposition illustrating restored marginal bone levels with stable peri‐implant soft tissues.

The clinical relevance of appropriate prosthetic component selection and EP design is illustrated by a representative clinical example (Figures 2, 3, 4, 5). Clinical and radiographic evaluation revealed that the pathology was localized at the interface between the prosthetic crown and a short titanium base, with a convex EP and excessive restorative angle (Figures 2a, 3a, 4b). The restoration was replaced with a monolithic zirconia crown featuring a concave, highly polished transmucosal contour, supported by a taller titanium base (2 mm) to relocate the prosthetic interface coronally (Figures 2b, 3b, 4c). Four months after prosthetic modification, the patient showed complete resolution of clinical inflammation and radiographic evidence of crestal bone remodeling (Figures 4d and 5). This case highlights how biologically guided prosthetic design—particularly EP geometry and abutment height—can restore peri‐implant tissue stability without surgical intervention when principles of soft tissue biology are respected (Figures 2, 3, 4, 5).

## 5 Provisionalization

The provisionalization phase plays a key role in the execution of a restoration which will promote biological stability. Also, provisionals will serve as a blueprint for the final restoration which is important especially in the esthetic area. The concepts of space, volume, and time have been described as critical perio‐prosthetic principles for optimizing peri‐implant tissue outcomes and stability [1]. Proper space created through the morphology of the provisional restoration defines the future soft tissue margin and interproximal architecture while allowing sufficient tissue thickness to prevent issues such as shimmering of the metal through mucosa [44]. Time in provisionalization is equally essential, as tissue maturation and remodeling occur during this stabilization phase. Milled poly‐methyl‐methacrylate (PMMA) is ideal for long‐term provisionals due to its mechanical strength, biocompatibility, and favorable esthetic properties [45]. In addition to milled PMMA, contemporary 3D‐printed provisional resins, including hybrid formulations with ceramic fillers, have been introduced as temporary materials [46].

The provisionalization strategy should be tailored to the clinical condition, whether the objective is margin preservation or margin re‐establishment and must consider the existing tissue phenotype as well as the magnitude and direction of tissue displacement [1]. When the goal is to maintain the current soft tissue margin, an immediate provisional placed at the time of implant insertion is preferred. This can be done by connecting the provisional restoration to the implant platform, or using an intermediate abutment respecting the “Restorative” and “Biological” rooms of the prosthetic framework [47]. In contrast, if soft tissue enhancement is required, a sub‐contoured provisional should be used to create space for grafted tissues, with the ideal contour subsequently re‐established to refine the esthetic outline [1, 26].

**FIGURE 6:**
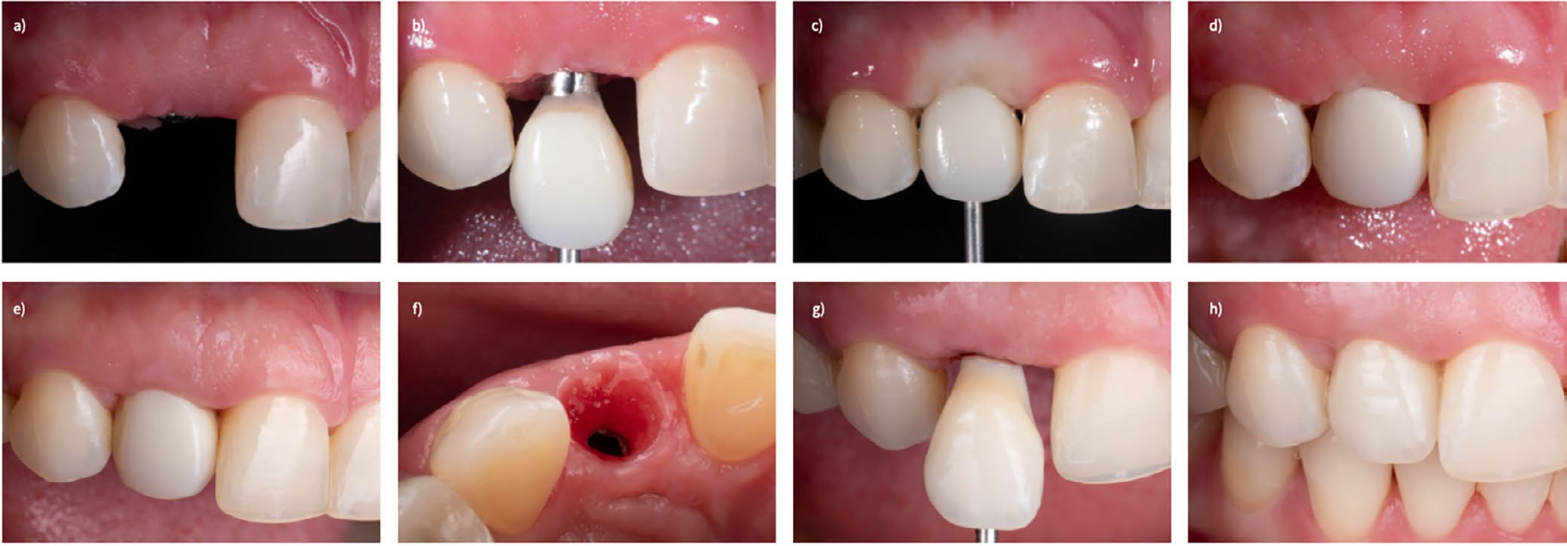
(a) Soft tissue margin resstablishment therapy needed on implant replacing the root of the maxillary right lateral incisor. (b) Full contour provisional with an active behavior is delivered to start tissue conditioning process. (c) Full contour provisional with active behavior, whose E‐zone exerts pressure on the soft tissue to establish a proper outline and support. (d) Open embrassures mesial and distal to full contour provisional. Adequate soft tissue volume present, and interproximal tissue creeping expected. (e) Interproximal tissue creeping can be seen after several weeks post‐op. Timing modifications and allowing tissues to mature in crucial to re‐establish the loss outlines. (f) A biologically‐thought provisional will condition the peri‐implant tissues adequately and promote health and future stability of the soft tissues. (g) Zirconia screw‐retained restoration is delivered with adequate contours and surface finishing. (h) Postdelivery image of final restoration, a balance between the soft tissue and the restoration is needed to achieve a proper esthetic outcome.

Margin reestablishment therapy is illustrated by a patient presenting for restorative refinement of a maxillary lateral incisor implant placed 6 months earlier (Figure 6). The site exhibited a slightly increased mesiodistal dimension and a flattened soft tissue architecture, while the tissue phenotype was thick and required only minimal apical displacement to achieve an optimal esthetic outcome. A prefabricated PMMA tooth was converted to an implant supported provisional crown using a pick‐up technique, with particular emphasis on defining line angles and interproximal contours to compensate for the increased space and promote favorable tissue adaptation. The cervical morphology of the provisional was carefully shaped to preserve embrasure integrity and facilitate coronal soft tissue migration. After a 6‐month provisionalization period allowing for tissue maturation and stabilization, the EP was transferred to the definitive restoration, and a screw‐retained zirconia crown was delivered. This case underscores the role of biologically guided provisional contours and adequate healing time in achieving stable peri‐implant soft tissue architecture, reinforcing the provisional restoration as a critical blueprint for the final prosthetic outcome (Figure 6).

In other scenarios, the provisional may be used to shape a healed ridge or re‐establish contours following healing with an abutment. Depending on the initial margin position, desired tissue displacement, site location, and phenotype, the clinician may need to gradually modify the contours to guide the soft tissue to its final architecture or, alternatively, fabricate a full‐contour provisional at once [1, 48, 49].

Although the definitive restoration often reproduces the EP of the provisional, this is not always the case. In immediate implant placement and soft‐tissue augmentation scenarios, provisionals are often intentionally under‐contoured to provide space for graft maturation and papilla formation. In such cases, the final restoration may incorporate refined contours, including controlled apical support or compression when indicated, to harmonize with the contralateral dentition.

Digital workflows now enable precise implant placement which will lead to adequate geometry of the EP [50]. Also, CAD/CAM abutments can replicate provisional contours, adjust emergence angles, and achieve highly polished surfaces (< 0.2 μm Ra) to reduce bacterial adhesion [51].

## 6 Discussion

Biologically driven prosthetic design is essential for long‐term peri‐implant tissue stability. Among design parameters, restorative angle and emergence contour are particularly influential. Histological evidence shows that respecting the vertical dimension of peri‐implant soft tissues, approximately 2 mm of junctional epithelium and 1.5–2 mm of connective tissue is fundamental to maintaining health [52].

Proper stability of the Implant Supracrestal Complex requires correct implant positioning, adequate soft‐tissue thickness and prosthetic contours that do not encroach upon the STH. Narrow restorative angles support epithelial organization, whereas wide convex angles may disrupt the seal and promote inflammation [9]. Excessive flaring can trigger connective‐tissue displacement or bone remodeling [40]. Taller titanium bases (≥ 2 mm) or custom abutments help relocate restorative margins coronally and preserve tissue integrity [19].

Modern CAD/CAM workflows enable the precise design of concave, polished transmucosal profiles (< 0.2 μm Ra) that support tissue stability and hygiene [51]. Convex profiles, in contrast, have been associated with mucosal recession [14, 15], mucositis [11], and peri‐implantitis [12]. While restorative angle clearly modulates local inflammation, disease progression appears multifactorial and depends on maintenance and host response.

No biologically validated thresholds for restorative angles currently exist. Reported values such as < 30° [12, 40] or < 40° [9, 14] are empirical. The European Federation of Periodontology (EFP) S3 guidelines (Recommendations 52–53) advocate cleansable contours but offer no quantitative criteria [53]. Both under‐ and over‐contouring increase plaque retention. Undercontouring may, however, be useful during provisionalization to accommodate soft‐tissue therapy before achieving the final shape.

**FIGURE 7:**
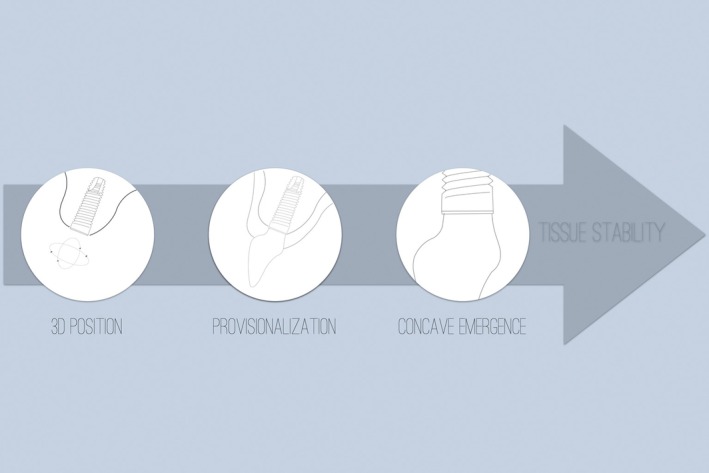
Fundamental steps for achieving long‐term peri‐implant tissue stability through biologically guided planning and clinical common sense. The sequence begins with proper three‐dimensional implant positioning, followed by the establishment of a biologically compatible emergence profile during the provisional phase, and finalized with a definitive restoration that maintains soft tissue support.

Ultimately, the anatomy and vascular requirements of the peri‐implant soft tissues must guide prosthetic design. When restorative margins or convex profiles invade the connective‐tissue zone, vascular supply may be compromised, leading to tissue collapse or recession [19, 20]. Biologically driven designs that respect this anatomy are essential for durable esthetic and functional outcomes (Figure 7).

As evidence expands, longitudinal studies are needed to establish biologically validated, standardized guidelines for emergence geometry and abutment height that balance esthetics, hygiene, and long‐term peri‐implant health.

## 7 Conclusions

Restorative angle and EP are critical in the long‐term stability of peri‐implant tissue health and esthetics. EPs that follow biological principles and clinical common sense—incorporating concave transmucosal contours, controlled restorative angles, and sufficient abutment height—support soft tissue integration and reduce the risk of plaque accumulation and inflammation. Advances in digital workflows have enabled precise replication of these biologically favorable designs, however, successful outcomes continue to rely on the clinician's understanding of peri‐implant anatomy and the application of sound judgment during treatment planning and execution. A biologically driven, patient‐specific approach remains fundamental for achieving predictable outcomes in implantology. Future longitudinal research should establish clear, quantitative parameters that integrate esthetic outcomes, plaque control, and peri‐implant tissue health.

## Funding

The authors have nothing to report.

## Disclosure

The authors have nothing to report.

## Conflicts of Interest

The authors declare no conflicts of interest.

## Data Availability

The data that support the findings of this study are available from the corresponding author upon reasonable request.
